# An Empirical Dilatancy Model for Coarse-Grained Soil under the Influence of Freeze–Thaw Cycles

**DOI:** 10.3390/ma15093167

**Published:** 2022-04-27

**Authors:** Yangsheng Ye, Degou Cai, Shuang Tian, Hongye Yan, Xianzhang Ling, Liang Tang, Yike Wu

**Affiliations:** 1 Railway Engineering Research Institute, China Academy of Railway Sciences Corporation Limited, Beijing 100081, China; yysh@rails.cn (Y.Y.); yanhongye_2005@163.com (H.Y.); 2 School of Civil Engineering, Harbin Institute of Technology, Harbin 150090, China; ts_hit@163.com (S.T.); xianzhang_ling@263.net (X.L.); tangliang@hit.edu.cn (L.T.); wyk_hit@163.com (Y.W.); 3 Chongqing Research Institute, Harbin Institute of Technology, Chongqing 401135, China

**Keywords:** freeze–thaw cycles, coarse-grained soil, dilatancy, empirical equations

## Abstract

In the era of high-speed trains, it is very important to ensure the safety and stability of rail tracks under adverse conditions including seasonal freezing and thawing. Freeze–thaw cycles (FTCs) affecting the engineering performance of coarse-grained soil (CGS) is one of the major reasons for track deterioration. The reported results of a number of static freeze–thaw triaxial tests on the shear behaviour of CGS are analysed herein. It was observed that confining pressure (*σ*_3_) and FTCs have a significant influence on the shear behaviour of CGS. In this paper, an empirical mathematical model has been proposed to capture the dilatancy of CGS subjected to FTCs during shearing. The empirical constants *a*, *b*, and *c* proposed in the model are a function of *σ*_3_ and FTCs. The results of the model have been compared with the laboratory experiments and are found to be in good agreement.

## 1. Introduction

Coarse-grained soil (CGS) has been widely used in the construction of high-speed railways due to its high shear strength and compaction capacity. A thorough understanding of the engineering properties of CGS is imperative in view of safety, stability and maintenance aspects. Dilation of CGS is important to capture the plastic nature of the material during shearing, and the proper understanding of the stress-dilatancy relationship is imperative for developing a constitutive model that correctly represents the plastic strains.

Systematic study of the relationship between dilatancy and friction angle yielded the simple, classical saw tooth model and the corresponding law of plastic flow [1,2,3]. Based on the principle of minimum energy ratio, Rowe [2] studied the deformation mechanism in CGS by considering a sliding motion between rigid cylindrical and spherical particles, and established the Rowe stress-dilatancy equation in terms of the stress and strain ratios. Furthermore, Wroth and Basset [4] argued that the expansion rate of soil is closely related to its current stress state. Bolton [5] proposed a new relative dilatancy index by investigating the shear capacity and dilatancy characteristics of various types of sandy soil. Houlsby [6] improved the modelled relationship between the dilatancy angle and the critical state line to characterise the influence of relative density on dilatancy. Mitchell and Soga [7] concluded that the stress-expansion relationship in CGS is greatly affected by factors such as soil structure and confining pressure. Despite significant progress having been made so far in the stress–dilatancy relationships of CGS, comprehensive research incorporating the freeze–thaw cycles (FTCs) effect is limited in a fundamental viewpoint. 

Past studies have shown that the engineering properties of CGS used for rail foundations in cold regions have been badly damaged by the FTCs, especially stress dilatancy behaviour [8,9]. A reduction in shear strength and dilation was reported for CGS subjected to FTCs [10,11,12]. This is why the design and maintenance of HSR requires an insightful understanding of the fundamental stress–strain response of track construction materials under complex thermo-mechanical conditions. Therefore, some efforts have been made to build a reliable stress dilatancy relationship of soils with incorporating the influence of FTCs to make constitutive models more accurately [13,14,15,16]. However, stress-dilatancy relationships incorporating the influence of FTCs are mainly limited to fine-grained materials, whereas stress-dilatancy relationships for CGM that capture the influence of FTCs are still in their infancy. Furthermore, the models in the current research models are too complex to use widely.

In this study, an empirical non-linear stress-dilatancy equation has been proposed to capture the dilatancy behaviour of CGS for a wide range of FTCs. In the models, the effects of FTCs and confining pressures (*σ*_3_) are examined, how they would affect the stress dilatancy relationship and are reflected through changes in the constants a, b and c. Finally, important conclusions and future work opportunities have been discussed.

## 2. Development of an Empirical Model for CGS under Different FTCs

Dilatancy is fundamental to the simulation of the stress-strain characteristics of soil. Based on the dilatancy equation proposed by Rowe [2], the dilatancy ratio ($D_{R}$) and the stress ratio (η) of granular materials can be calculated using Equations (1) and (2), respectively [17].

(1) $$D_{R}=1-\frac{{d\varepsilon }_{v}}{{d\varepsilon }_{1}}$$

(2) $$\eta =\frac{q}{p}$$

where ${d\varepsilon }_{v}$ and ${d\varepsilon }_{1}$ denote the volumetric strain increment and the axial strain increment, respectively, and *q* and *p* denote deviatoric stress and average stress, respectively.

In this study, the dilatancy characteristics of CGS were further analysed by investigating the dependence of the stress ratio on the dilatancy ratio. Considering the nonlinear behaviour of CGS during the shear process, an empirical dilatancy model for CGS considering FTC, given by Equation (3), was established based on the data collected during the triaxial tests to capture the dilatancy behaviour of CGS under FTCs.

(3) $$\frac{q}{p}=a{\left[1-\frac{{d\varepsilon }_{v}}{{d\varepsilon }_{1}}\right]}^{2}+b\left[1-\frac{{d\varepsilon }_{v}}{{d\varepsilon }_{1}}\right]+c$$

where *a*, *b*, and *c* denote empirical constants that depend on the confining pressure and the number of FTCs. It is noted that albeit there are no clear physical concepts for the above-mentioned parameters, a simple and practical nonlinear dilatancy model has been initially proposed following some previous studies [18,19,20,21].

## 3. Results and Discussion

### 3.1. Static Freeze–Thaw Triaxial Test

**Figure 1:**
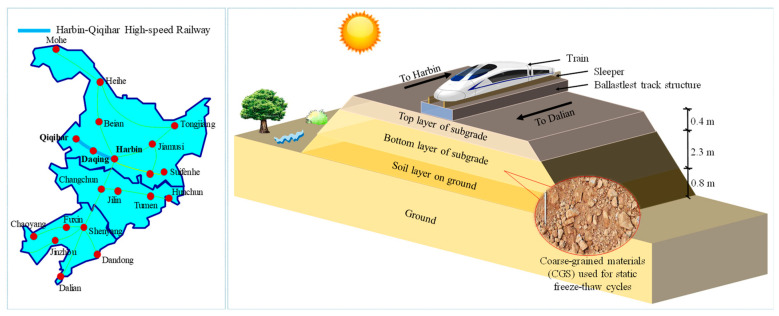
The schematic construction of the Harbin-Qiqihar high-speed railway in Northeast China.

Static freeze–thaw triaxial tests were conducted at Shijiazhuang Tiedao University to elucidate the static properties of CGS under different FTCs and confining pressures (*σ*_3_) from 30–90 kPa in the low-temperature triaxial laboratory [22]. The CGS used under Harbin-Qiqihar high-speed railway line in Northeast China was used as the test soil (see Figure 1). FTCs were varied from 0 to 10 cycles to represent different field conditions. The triaxial test specimen was 100 mm in diameter and 200 mm in height. More details of the materials and testing procedures can be found elsewhere (e.g., Ling et al. [22], Tian et al. [23]). The stress–strain and volume change behaviour during drained tests at varying FTCs and *σ*_3_ are considered here.

**Figure 2:**
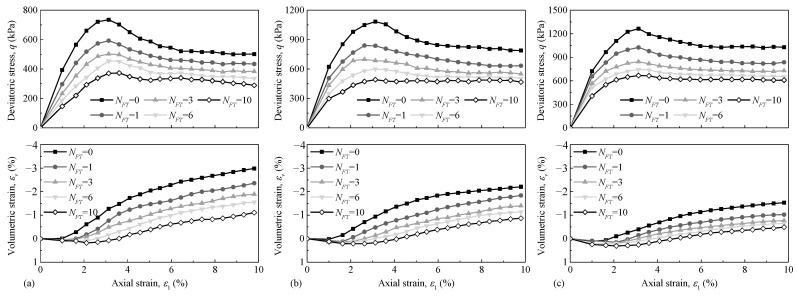
Dependence of deviatoric stress and volumetric strain on axial strain of tested CGS corresponding to different numbers of FTCs (modified after Ling et al. [18]): (**a**) *σ*_3_ = 30 kPa, (**b**) *σ*_3_ = 60 kPa, and (**c**) *σ*_3_ = 90 kPa.

Figure 2 shows the typical variation of deviator stress and volumetric strain with axial strain at different FTCs and *σ*_3_. It is evident that the addition of FTCs has a significant influence on the shear and volumetric strain behaviour of CGS. In general, when *σ*_3_ and FTC increase, the initial compression increases. This is because of an increasing void space after freeze–thaw cycles which allows the specimens to compress. However, the FTCs do not seem to change the overall trends of the stress–strain curves.

### 3.2. An Empirical Dilatancy Model for CGS Considering FTCs 

The values of *a*, *b*, and *c* under different confining pressures and numbers of FTCs are listed in Table 1. The software used to fit the curve in the current study is Microsoft Excel 2019.

**Figure 3:**
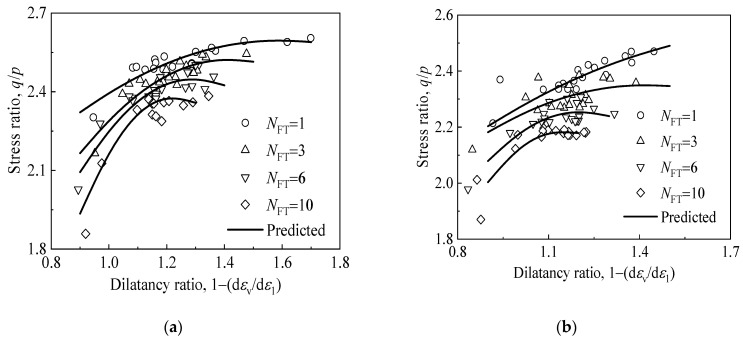

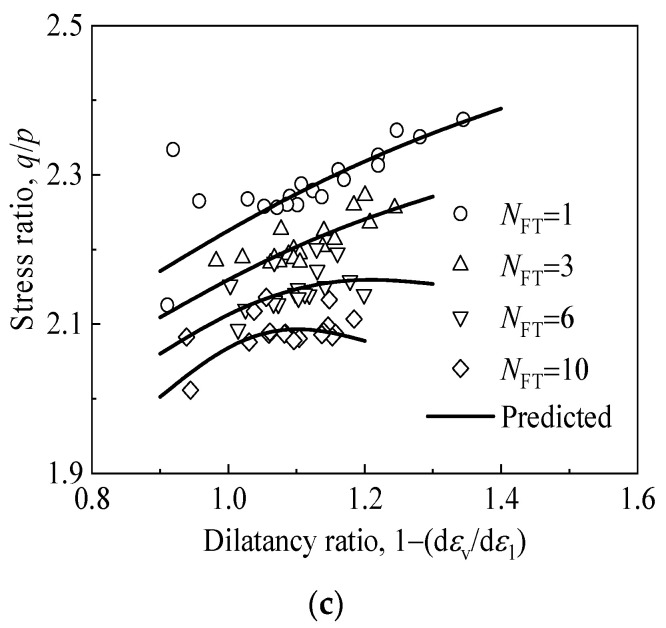
Relationship between the stress ratio and dilatancy ratio of CGS corresponding to different numbers of FTCs and confining pressures: (**a**) *σ*_3_ = 30 kPa, (**b**) *σ*_3_ = 60 kPa, and (**c**) *σ*_3_ = 90 kPa.

As depicted in Figure 3, the stress ratio of CGS increased nonlinearly with an increase in the dilatancy ratio under the influence of FTCs. Under the same confining pressure, the stress ratio of CGS decreased with an increase in the number of FTCs, and the variation range and maximum value of the dilatancy ratio decreased with an increase in the number of FTCs. For the same number of FTCs, the stress ratio of CGS decreased with an increase in confining pressure, and the variation range and maximum value of the dilatancy ratio decreased with an increase in confining pressure.

The decrease in stress ratio and dilatancy ratio with the increase in the number of FTCs and confining pressure may be primarily attributed to the following factors. (1) FTCs exert a deteriorating effect on CGS—the modulus and strength of CGS decrease with an increase in the number of FTCs. In addition, after several FTCs, the internal structure of the originally dense CGS changes, and the number of pores between the soil particles are increased, which weakens the dilatancy property of CGS. (2) The mechanical properties of CGS are significantly affected by confining pressure—an increase in confining pressure exerts an inhibitory influence on the dilatancy of CGS.

As listed in Table 1, the value of the empirical constant, *c*, under identical confining pressures and different numbers of FTCs, was close to 0 in most cases, either positive or negative. As such data may exhibit significant fluctuations during the subsequent normalisation process, the empirical constant *d* = *c* + 10 was defined for the subsequent analysis and it was used to represent the variation rule of *c*. Furthermore, $A\cdot \exp(B\cdot \sigma_{3}$) was selected as the normalised factor under different confining pressures. The average empirical constants *a*, *b*, and *d* can be calculated using Equations (4)–(6).

(4) $$a_{avg}=\frac{1}{n}\overset{n}{\underset{i=1}{\sum }}a_{i}=\alpha \cdot \exp(\beta \cdot \sigma_{3})$$

(5) $$b_{avg}=\frac{1}{n}\overset{n}{\underset{i=1}{\sum }}b_{i}=\gamma \cdot \exp(\delta \cdot \sigma_{3})$$

(6) $$d_{avg}=\frac{1}{n}\overset{n}{\underset{i=1}{\sum }}d_{i}=\zeta \cdot \exp(\eta \cdot \sigma_{3})$$

where $a_{avg}$, $b_{avg}$, and $d_{avg}$ denote the average empirical constants *a*, *b*, and *d*, respectively; *n* = 4 corresponds to 1, 3, 6, and 10 FTCs; and α, β, γ, δ, ζ, and η denote undetermined coefficients whose values are presented in Table 2. The values of $a_{avg}$, $b_{avg}$ and $d_{avg}$ under specific confining pressures can be calculated using Equations (4)–(6).

By fitting Equations (7)–(9), the normalised empirical constants *a*, *b*, and *d* corresponding to different numbers of FTCs can be obtained.

(7) $$\frac{a_{i}}{a_{avg}}{=M}_{1}\cdot {\exp(N}_{1}\cdot N_{\mathrm{FT}})$$

(8) $$\frac{b_{i}}{b_{avg}}{=M}_{2}\cdot {\exp(N}_{2}\cdot N_{\mathrm{FT}})$$

(9) $$\frac{d_{i}}{d_{avg}}{=M}_{3}\cdot {\exp(N}_{3}\cdot N_{\mathrm{FT}})$$

where $M_{1}$, $M_{2}$, $M_{3}$, $N_{1}$, $N_{2}$, and $N_{3}$ denote undetermined coefficients whose values are listed in Table 2.

**Figure 4:**
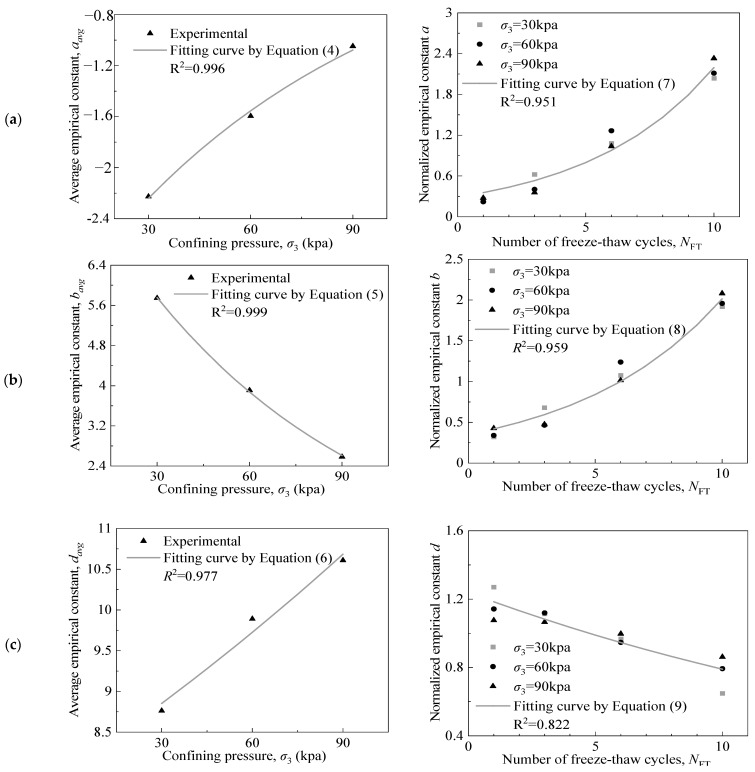
Fitting curves of (**a**) empirical constant *a*; (**b**) empirical constant *b*; and (**c**) empirical constant *d*.

Figure 4 depicts the fitting curves of the average empirical constants, $a_{avg}$, $b_{avg}$ and $d_{avg}$, and the normalised empirical constants, *a*, *b*, and *d.* Under different confining pressures, both the normalised empirical constants, *a* and *b*, increased nonlinearly and the normalised empirical constant, *d*, decreased nonlinearly with an increase in the number of FTCs. After 6–10 FTCs, the physical and mechanical properties of CGS tended to stabilise. However, the normalised empirical constants *a*, *b*, and *d* did not stabilise even after 6–10 FTCs. The rate of change of the normalised empirical constants, *a* and *b*, increased with an increase in the number of FTCs, while that of the normalised empirical constant, *d*, remained nearly constant.

As given by Equations (10)–(12), $a_{n}$, $b_{n}$, and $c_{n}$ can be obtained by combining Equation (4) and Equation (7), Equation (5) and Equation (8), and Equation (6) and Equation (9), respectively.

(10) $$a_{n}=\alpha \cdot M_{1}\cdot \exp(\beta \cdot \sigma_{3}{+N}_{1}\cdot N_{\mathrm{FT}})$$

(11) $$b_{n}=\gamma \cdot M_{2}\cdot \exp(\delta \cdot \sigma_{3}{+N}_{2}\cdot N_{\mathrm{FT}})$$

(12) $$c_{n}=\zeta \cdot M_{3}\cdot \exp\eta \cdot \sigma_{3}{+N}_{3}\cdot N_{\mathrm{FT}}-10$$

By substituting Equations (10)–(12) into Equation (3), Equation (13) was obtained, which can be used to predict the relationship between the stress ratio and dilatancy ratio corresponding to any confining pressure and any number of FTCs.

(13) $$\frac{q}{p}=\alpha \cdot M_{1}\cdot \exp(\beta \cdot \sigma_{3}{+N}_{1}\cdot N_{\mathrm{FT}}){\left[1-\frac{{d\varepsilon }_{v}}{{d\varepsilon }_{1}}\right]}^{2}+\gamma \cdot M_{2}\cdot \exp(\delta \cdot \sigma_{3}{+N}_{2}\cdot N_{\mathrm{FT}})\left[1-\frac{{d\varepsilon }_{v}}{{d\varepsilon }_{1}}\right]+\zeta \cdot M_{3}\cdot \exp(\eta \cdot \sigma_{3}{+N}_{3}\cdot N_{\mathrm{FT}})-10$$

### 3.3. Validation of the Proposed Equation with the Literature

**Figure 5:**
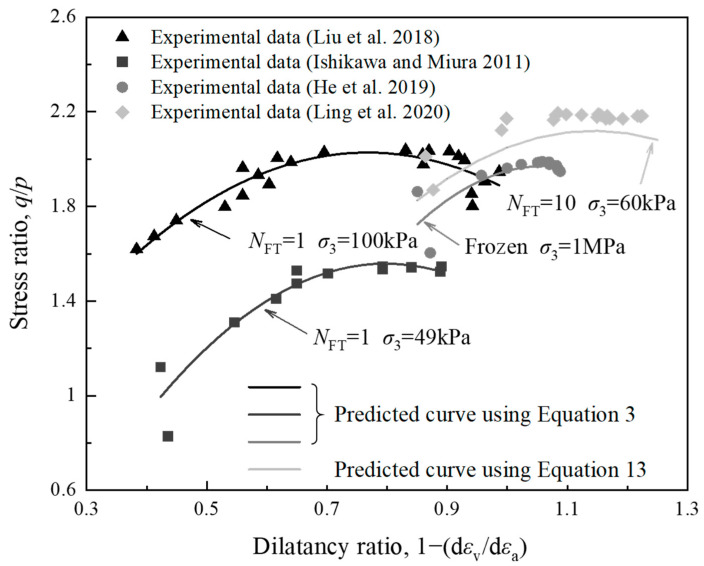
Comparison between predicted and experimental values of stress ratio and dilatancy ratio.

Owing to the lack of sufficient data, the models given by Equations (3) and (13) were verified based on only a small sample of experimental data. To this end, the previously reported triaxial test results of four different types of soil—China’s tailing soil [24], Japan’s volcanic CGS [25], China’s frozen sand [26], and China’s CGS for high-speed railway subgrade [22]—were considered. Figure 5 depicts a comparison between the triaxial test results and the predicted curves obtained using the proposed model corresponding to the four types of soil. Table 3 was used to computing the constants *a*, *b*, and *c* for validating the experimental data. The empirical constants *a*, *b* and *c* were found to be −2.892, 4.44, and 0.324, respectively, for tailing soil subjected to 1 FTC, which were used to predict the experimental data reported by Liu et al. [24]. Whereas, *a* = −4.017, *b* = 6.409, and *c* = −0.998 was used to predict the experimental data reported by Ishikawa and Miura [25] for volcanic coarse-grained soil subjected to 1 FTC. Furthermore, the experimental data for sandy soil in frozen state and CGS subjected to 1 FTC reported by He et al. [26] and Ling et al. [22], respectively, were also used to validate the proposed model. The empirical constants a, b and c were found to be −5.514, 11.71, −4.246 and −3.408, 7.797, −2.339, respectively. It is evident from Figure 5 that the proposed equations (Equations (3) and (13)) have shown a very good agreement with the past experimental data.

## 4. Conclusions

Freezing-thawing cycles (FTCs) due to seasonal variations may induce instability in the railway track. Static freeze-thaw triaxial tests on coarse-grained soils (CGS) demonstrated a reduction in the shear strength and dilation upon shearing and further exhibited a non-linear relationship between the stress ratio and dilatancy ratio. Therefore, empirical dilatancy models of CGS were proposed to address the effects of FTCs and confining pressure (*σ*_3_). From the obtained results and analyses presented in this study, the main conclusions are drawn as follows:

(1) An empirical dilatancy model of CGS considering FTCs was proposed to represent the dilatancy of CGS. The relationship between the stress ratio and the dilatancy ratio can be well-captured by a second-order polynomial fitting. The values of the empirical constants *a*, *b* and *c* introduced in this model depend on the FTCs and *σ*_3_.
(2) The best fit *a* and *c* values increase in the form of an exponential function with increasing *σ*_3_, while *b* values exhibit the opposite trend. On the other hand, the changing law of the *a* and *b* values increase with increasing FTCs following a form of an exponential function. Meanwhile, c values exhibit the opposite trend. It is interesting to note that the *a*, *b*, and *c* values vary between −0.37 and −4.53, 1.82 and 11.03, −4.32 and 1.41, respectively.
(3) The prediction performance of the proposed model was verified using experimental data, and the validity and applicability of the proposed model was validated. The minimum value of the biggest determination coefficient R^2^ is 0.816 except for sandy soil under frozen state, which show that the proposed non-linear stress-dilatancy equation capture the test data well for the FTC conditions.
(4) Further research is needed to address the limitations in the present study; for instance, certain aspects related to stress-induced anisotropy, the effects of creep, long-term cyclic loading and the influence of particle breakage will need to be investigated in the future as an extension of the proposed model.

## Figures and Tables

**Table 1:** Empirical Constants, *a*, *b*, and *c*.

Confining Pressure, *σ*_3_ (kPa)	FTC, *N*_FT_ (−)	*a*	*b*	*c*	*R^2^*
30	1	−0.5822	1.8480	1.1303	0.825
	3	−1.3870	3.9102	−0.2301	0.808
	6	−2.4030	6.1883	−1.5292	0.869
	10	−4.5342	11.0304	−4.3194	0.909
60	1	−0.3523	1.3240	1.2973	0.856
	3	−0.6427	1.8171	1.0671	0.543
	6	−2.0180	4.8403	−0.6425	0.823
	10	−3.3690	7.6584	−2.1612	0.845
90	1	−0.2907	1.1040	1.4135	0.539
	3	−0.3732	1.2260	1.3086	0.875
	6	−1.0870	2.6253	0.5784	0.786
	10	−2.4421	5.3794	−0.8606	0.899

**Table 2:** Regression coefficients for model parameters.

Fitting Equations	Regression Coefficients	Values
$a_{avg}=\frac{1}{n}\sum_{i=1}^{n}a_{i}=\alpha \cdot \exp\left(\beta \cdot \sigma_{3}\right)$	α	−3.2310
	β	−0.0122
$b_{avg}=\frac{1}{n}\sum_{i=1}^{n}b_{i}=\gamma \cdot \exp\left(\delta \cdot \sigma_{3}\right)$	γ	8.5480
	δ	−0.0132
$d_{avg}=\frac{1}{n}\sum_{i=1}^{n}d_{i}=\zeta \cdot \exp\left(\eta \cdot \sigma_{3}\right)$	ζ	8.0590
	η	0.0031
$\frac{a_{i}}{a_{avg}}=M_{1}\cdot \exp\left(N_{1}\cdot N_{FT}\right)$	$M_{1}$	0.2898
	$N_{1}$	0.2024
$\frac{b_{i}}{b_{avg}}=M_{2}\cdot \exp\left(N_{2}\cdot N_{FT}\right)$	$M_{2}$	0.3517
	$N_{2}$	0.1745
$\frac{d_{i}}{d_{avg}}=M_{3}\cdot \exp\left(N_{3}\cdot N_{FT}\right)$	$M_{3}$	1.2390
	$N_{3}$	−0.0451

**Table 3:** Values of the parameters of the prediction model.

Source of Experimental Data	Material	FTC	Confining Pressure, *σ*_3_ (kPa)	a $\cdot (a_{n})$	b $\cdot (b_{n})$	c $\cdot (c_{n})$	*R* ^2^
Liu et al. [16]	Tailing soil	N_FT_ = 1	100	−2.892	4.440	0.324	0.816
Ishikawa and Miura [17]	Volcanic coarse-grained soil	N_FT_ = 1	49	−4.017	6.409	−0.998	0.889
He et al. [18]	Sandy soil	Frozen	1000	−5.514	11.710	−4.246	0.633
Ling et al. [14]	Coarse-grained soil	N_FT_ = 1	60	−3.408	7.797	−2.339	0.845

## Data Availability

Not applicable.
